# Effectiveness of multiple botulinum toxin sessions and the duration of effects in spasticity therapy in children with cerebral palsy

**DOI:** 10.1007/s00381-018-3923-6

**Published:** 2018-07-30

**Authors:** Anna Mirska, Wojciech Kułak, Bożena Okurowska-Zawada, Elżbieta Dmitruk

**Affiliations:** Department of Pediatric Rehabilitation and Center of Early Support for Handicapped Children “Give a Chance”, Medical University of Bialystok, Waszyngtona 17, 15-274 Białystok, Poland

**Keywords:** Cerebral palsy, Botulinum toxin, Muscle tone, Range of motion, Gait

## Abstract

**Purpose:**

The aim of this study was to assess the effectiveness of long-term therapy with multiply botulinum neurotoxin (BoNT) injections.

**Methods:**

In 2004–2010, 60 children with spastic cerebral palsy aged 2–16 were treated multiple botulinum toxin sessions (injections in gastrocnemius muscle and soleus muscles). In each patient, we rated muscle tone by Modified Ashworth Scale, passive range of motion in ankle joint with extended, and flexed knee joint and gait using the Physician Rating Scale. Assessment was done before and after injection, up to eight BoNT sessions.

**Results:**

The generalized additive models showed that a single treatment effect was visible for 3 months. The number of injections did not impact the effectiveness. Improvement in muscle tone was greater in children with hemiplegia than diplegia (*β* = − 0.294; *p* = 0.014). Improvement in range of motion with extended knee joint was greater in hemiplegic than diplegic types (*β* = 0.414; *p* =0.002), and improvement in range of motion with flexed knee was greater in children with more severe impairment (Gross Motor Function Classification System III vs. I, *β* = 0.0603, *p* = 0.025; V vs. I, *β* = 0.691, *p* = 0.023). The gait improvement rate decreased with patient age (*p* = 0.007).

**Conclusions:**

BoNT therapy is effective regardless of the number of injection sessions and duration of treatment. However, it is affected by the patient’s age, type of cerebral palsy, and degree of impairment.

## Introduction

Cerebral palsy is a group of permanent disorders in movement and posture development that limits physical activity. It is caused by disruptions in brain development during pregnancy and infancy. Motor disorders are often accompanied by sensory disturbance, cognitive and perception disorders, difficulties in communication, behavior disorders, epilepsy, and secondary issues of the musculoskeletal system. Spasticity is the most common symptom. It initially impairs motor functions and gradually contributes to osteo-articular malformation [1].

Botulinum neurotoxin (BoNT) is a spasticity treatment in children with cerebral palsy [2]. Intramuscular injections inhibit secretion of acetylcholine by neuromuscular synapses. In effect, the muscle is not stimulated, and thus muscle tone decreases [3]. This enables the subject to overcome some spasticity consequences. BoNT treatment is often applied long term. It is recommended to start this in the second year of life and to continue to age 8–10 years via multiple injections. However, it should not be applied more than once every 3 months because secondary resistance and overlaying dose effects may appear [4].

The efficacy of BoNT therapy is affected by the dose, administration, patient age, and type of parallel rehabilitation [5–7]. Long-term studies comparing the effects of BoNT treatment after each injection are rare [8]. Previous studies have shown that BoNT therapy is effective and therefore is currently a common clinical procedure in cerebral palsy [4–6]. However, there is a need to investigate if multiply injections weaken the therapeutic effects as well as to identify the optimal interval between injections to maintain the best possible improvement. The aim of this study was to assess the effectiveness of long-term therapy with multiply BoNT injections including the time of observed improvement.

## Methods

### Patients

From 2004 to 2010, 60 patients aged 2 to 16 (5.8 ± 3.4) with spastic cerebral palsy were enrolled. Thirty were diagnosed as tetraplegic, 20 diplegic, and 10 hemiplegic. Patients represented different levels of impairment on the Gross Motor Function Classification System (GMFCS) [9]. BoNT (Dysport) was injected into the gastrocnemius and soleus muscles with an average dose of 13.2 j/kg/mc (± 3.85) as provided for in the national drug program. Over these 6 years, the patients were injected between one and eight times with intervals of 3–66 months (10 months on average) (Table 1).

**Table 1:** Clinical characteristics of 60 patients with cerebral palsy

Characteristic	Value
Age	
Mean	5.8 ± 3.4
Range	2–16
Sex	
Male	33
Female	27
Type of spastic cerebral palsy	
Hemiplegia	10
Diplegia	20
Tetraplegia	30
GMFCS level	
I	3
II	27
III	10
IV	13
V	7
Number of BoNT treatments	
1	37
2	19
3	13
4	13
> 5	18

*GMFCS* Gross Motor Function Classification System, *BoNT* Botulinum Neurotoxin

### Patient’s assessment

The following features were measured in each patient: muscle tone was assessed with the Modified Ashworth Scale (MAS) [10], gait was assessed with the Physician Rating Scale (PRS) [11], and passive range of motion was assessed with the ankle joint with extended (ROM-E) and flexed knee joint (ROM-F). ROM was assessed from 0 to 3. If flex was not achieved at all, then the patient was scored with 0 points. If there was a difference between the base and the final position fitted 0°–10° range, then it was scored 1; 10°–20° was scored 2; and > 20° was scored 3. According to the study protocol, patients were rated before and 2 weeks, 6 weeks, and 3 months after injection. In the case of multiple injections, the “before” assessments of the following injections were used to study the duration of therapy improvement (maximally up to 18 months) after previous injection. At that time, all patients were receiving NDT-Bobath rehabilitation, thermotherapy and electrotherapy.

To analyze the effectiveness of the BoNT injections, MAS, PRS, ROM-E, and ROM-F results were measured in the next therapy session, which was 3 to 12 months after the prior injection. In order to check, the effects of multiple injections, data from examination at 2 weeks after each treatment, were only included in this investigation (because the previous study indicated that the improvement was most pronounced during this period) [12]. A similar approach was used to test the relationship between therapy effect and patient age.

### Statistical analysis

The generalized additive model (GAM) was used to test if/how improvement changed as a function of multiple injections of BoNT. The variables included time elapsed from injection, patient’s age, and the number of injections. Link functions and distribution type of the models was decided via the Akaike information criterion (AIC). Models were built with a Gaussian distribution and “identity” link function. These iterations were conducted by adding other variables (type of cerebral palsy and GMFCS level) to assess their influence on improvement gained. Analyses used R software including version 3.3.2 with the “mgcv” package.

## Results

**Fig. 1:**
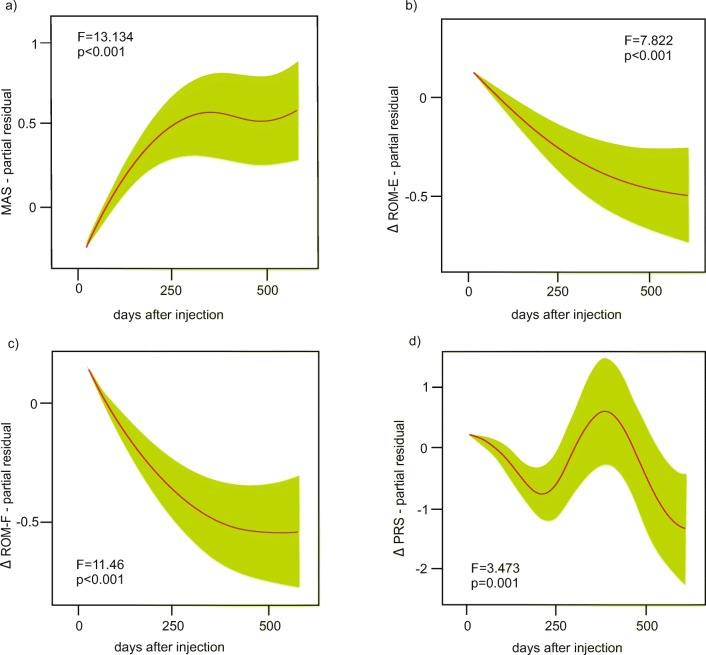
Generalized additive model models of temporal dynamics of MAS (Modified Ashworth Scale) (**a**), ROM-E (range of motion in ankle joint with extended knee joint) (**b**), ROM-F (range of motion in ankle joint with flexed knee joint) (**c**), and PRS (Physician Rating Scale) (**d**); Δ is the difference between post-injection and pre-injection scores

The time elapsed since BoNT injection significantly influenced the change in all features. The change in MAS increased with time while change in ROM-E and ROM-F decreased (Fig. 1). The GAM models showed that the parameters improved up to 3 months after injection, and there was a decline after this point. Stabilization was achieved 10 months from injection.

**Fig. 2:**
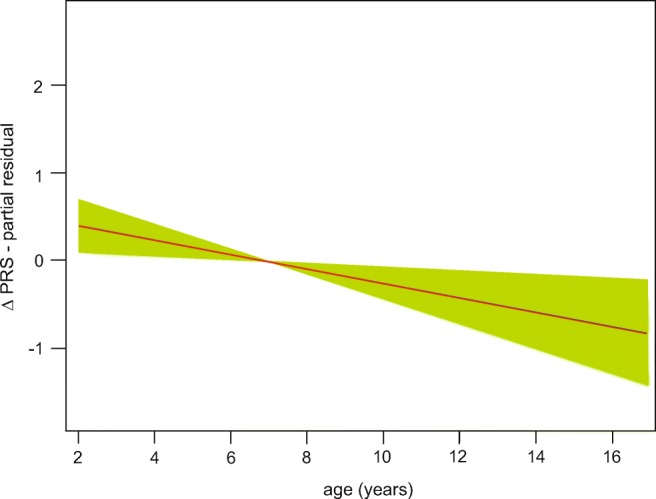
Generalized additive model of change in Physician Rating Scale (PRS) with patient’s age. Δ is the difference between post-injection and pre-injection scores

The number of injections did not affect the outcome parameters (Table 2). Only the change in PRS was significantly affected by patient age. The observed changes were positively affected by age up to 7 years. Beyond this, age was negatively correlated to therapy improvement (Fig. 2).

**Table 2:** General additive model results for clinical assessments used to study the effectiveness of botulinum neurotoxin (BoNT) treatment after 2 weeks at the injection

Parameter	Observed change (mean; ±)	Two-factor models				Four-factor models, including effects of type of cerebral palsy and GMFCS level			
		Number of BoNT treatments		Patient’s age		Number of BoNT treatments		Patient’s age	
		F	*p*	F	*p*	F	*p*	F	*p*
∆ MAS	− 0.49 ± 0.44	0.127	0.722	0.467	0.6	0.169	0.682	0.101	0.744
∆ ROM-E	0.36 ± 0.48	1.02	0.385	1.139	0.288	2.244	0.156	0.234	0.63
∆ ROM-F	0.37 ± 0.44	0.79	0.38	1.425	0.206	1.067	0.275	1.2387	0.278
∆ PRS	0.90 ± 1.15	0.022	0.884	7.491	0.007	0.043	0.836	6.083	0.016

*MAS* Modified Ashworth Scale, *ROM-E* passive range of motion in ankle joint with extended knee joint, *ROM-F* passive range of motion in ankle joint with flexed knee joint, *PRS* Physician Rating Scale; ∆ is the difference between post-injection and pre-injection scores

The type of cerebral palsy as well as impairment level (in GMFCS) influenced the assessments (Table 3). The change in MAS values were lower and higher in ROM-E in hemiplegic vs. diplegic type of cerebral palsy. Scores for hemiplegic patients were improved by 0.294 in MAS and 0.414 in ROM-E compared to diplegic ones. The changes in ROM-F were positively affected by higher than I GMFCS levels: patients scored on levels III and V of GMFCS obtained better results by 0.603 and 0.691, respectively. Change in PRS values were not affected by the variables tested.

**Table 3:** Generalized additive model significance test results for changes in values of clinical assessments after BoNT treatments (2 weeks after the injection) were tested as a function of cerebral palsy type and impairment level

Variables	Regression parameter	SE	*t*	*p*
∆ MAS				
Intercept	− 0.776	0.23	− 3.38	0.001
Hemiplegic vs. diplegic	− 0.294	0.118	− 2.499	0.014
∆ ROM-E				
Intercept	− 0.077	0.25	− 0.307	0.76
Hemiplegic vs. diplegic	0.414	0.13	3.172	0.002
∆ ROM-F				
Intercept	0.005	0.237	0.022	0.983
GMFCS level III vs. GMFCS level I	0.603	0.264	2.286	0.025
GMFCS level V vs. GMFCS level I	0.691	0.298	2.321	0.023

*MAS* Modified Ashworth Scale, *ROM-E* passive range of motion in ankle joint with extended knee joint, *ROM-F* passive range of motion in ankle joint with flexed knee joint, *GMFCS* Gross Motor Function Classification System; ∆ is the difference between post-injection and pre-injection scores

## Discussion

Of the numerous papers focusing on BoNT efficacy, only a few dealt with multiple injections over long-term therapy. In fact, most clinical therapies for spasticity in children with cerebral palsy are conducted in this way. Therefore, the questions raised at the beginning of this paper are relevant to clinical practice.

We have shown that the time elapsed from BoNT injection significantly affected its efficacy. The GAM model showed a gain in tested features by 3 months similar to the results obtained by other authors. The decrease in muscle tone after therapy is known to last for 12 weeks [5, 13], 16 weeks [14, 15], or even up to 6 months [16–18]. However, not all studies have confirmed this last finding [15, 19]. The improvement in the range of motion [13, 15, 16, 20] as well as in motor functions [15, 17, 19, 20] is a secondary effect of BoNT treatment; this lasts for about 6 months. The models built on clinical data indicate that after improvement in these traits, there is eventual worsening and finally stabilization. After improvement, muscle tone gradually increased up to 10–12 months from injection, while the range of motion in ankle joint began to decrease up to around 16 months.

The primary effects of BoNT therapy are associated with a decrease in muscle tone that is shorter than the secondary effects. Therefore, there are improved motor functions including gait that may last longer [20–22]. In our study, improvement in PRS lasted for 3 months; however, there is still early oscillation (Fig. 1) for reasons that are unclear. PRS assessment involves setting foot in the stance phase of gait cycle and is an accurate tool along with the other parameters used here. Moreover, PRS components like speed of gait, knees position during gait, and degree of crouch, we did not register changes. This would significantly influence the final PRS assessment and also the improvement rate curve. The accuracy of the assessment might be influenced by the fact that it was assessed in real time. The best assessment is supposed to be conducted through split-screen video in a slow-motion facility together with the use of a modified PRS version [3].

The literature shows that each BoNT injection causes desirable pharmacological effect, which decreases muscle tension [23–25]. Tedroff et al. found that BoNT may be effective for long-term spasticity reduction, but not in the prevention of contracture development. They also showed that the best gain in ROM was achieved after the first injection. After subsequent injections, the effect of the increasing range of motion was lower and insignificant [23]. Better improvement after the first injection was also observed by Linder et al. [24] and Fattal-Valevski et al. [25]; however, assessments of motor functions with the GMFM test showed identical improvements.

Higher and statistically significant gain was noted after the first injection in studies by Papavisilou et al. [26] and Fattal-Valevski et al. [25]. Also, in study by Read et al., a statistically significant gait improvement was especially marked after the first treatment and the effect was maintained also after two following treatments [27]. In addition, Hong et al. found that gait improved more in children with two or fewer injections than in patients with more than two injections [28]. A meta-analysis conducted by Kahraman et al. using studies from 1990 to 2015 showed that the first two injections let to functional improvement in children with spasticity. Unfortunately, they could not compare the effectiveness of more than one therapy repetition due to methodological incompatibilities of the studies included [8]. Our study showed that the number of injections did not affect improvement rate of the tested parameters. Although the sample size differed in groups with different numbers of injections as well as those that differed in the interval between injections.

Only gait had a significant improvement with patient’s age. The increase in gait declined with age up to 7 years, worsened further after that (Fig. 2). This is because motor function development in children with cerebral palsy lasts through age 7 [29]; nearly 90% of motor skills are already achieved by age 5 [30]. Therefore, children between the 1st and 5th year are expected to best respond to BoNT therapy [3], and early age injections help to develop less-pathological gait patterns that control groups [31, 32]. Wissel et al. obtained similar results. Here, the best gain was achieved in children below 7 years old both in gait and muscle tone [33].

The other parameters assessed were not affected by patient’s age, but some studies found a relation with passive dorsiflexion in ankle joint [34, 35]. This might be because younger children have more spastic muscles than those with a fixed contracture [36]. Eames et al. suggested that BoNT treatment efficacy is no longer by patient age once a fixed contracture appeared in the muscle [37]. The most effective approach would therefore be to start BoNT therapy while the muscle is still in a dynamic contracture phase. The same authors have suggested that the frequency of structural modifications increase with patient’s age; this decreases therapy efficacy in older children [37]. However, this does not mean that BoNT therapy is useless in improving the range of motion in older children [38, 39].

We found other factors that influence the effectiveness of therapy including type of cerebral palsy and motor impairment level. Children with hemiplegia had better improvement in muscle tone as well as in range of motion than children with diplegia. Moreover, children with higher than type I GMFCS level showed a greater gain in their range of motion. Other studies on BoNT treatment in cerebral palsy did not confirm the impact of cerebral palsy type nor impairment level in gross motor functions for muscle tone or range of motion [40, 41]. However, Fazzi et al. did show that these factors affected gait quality (PRS) and selective motor control [41]. Both outcomes were better in children with hemiplegia and milder motor impairment.

## Conclusion

Our study showed BoNT therapy is effective in children with cerebral palsy regardless of the number of sessions. Best results were achieved in children under age 7 with hemiplegia and greater impairment than level I on the GMFCS scale. The treatment gain was highest up to 3 months after injection. Therefore, BoNT therapy can be safely and effectively repeated every 3–6 months.
